# “Dance Well”—A Multisensory Artistic Dance Intervention for People with Parkinson’s Disease: A Pilot Study

**DOI:** 10.3390/brainsci15040357

**Published:** 2025-03-29

**Authors:** Daniele Volpe, Maria Giulia Baldassarre, Leila Bakdounes, Maria Concetta Campo, Davide Ferrazzoli, Paola Ortelli

**Affiliations:** 1 Department of Neurorehabilitation, Fresco Parkinson Excellence Center, “Casa di Cura Villa Margherita”, S. Stefano Riabilitazione, 36057 Arcugnano, Vicenza, Italy; baldi.gmp@hotmail.it (M.G.B.); leila.bakdounes@kosgroup.com (L.B.); mariaconcetta.campo@gmail.com (M.C.C.); 2 Dance Well Project Bassano del Grappa, 36061 Bassano del Grappa, Vicenza, Italy; 3 Department of Neurorehabilitation, Hospital of Vipiteno-Sterzing (SABES-ASDAA), Teaching Hospital of the Paracelsus Medical Private University (PMU), 39049 Vipiteno-Sterzing, Bolzano, Italy; davide.ferrazzoli@sabes.it (D.F.); paola.ortelli@sabes.it (P.O.)

**Keywords:** Parkinson’s disease, motor–cognitive interplay, neurorehabilitation, dance, art

## Abstract

**Background/Objectives**: Parkinson’s disease (PD) is a complex neurodegenerative disorder responsible for both motor and non-motor disturbances impairing motor behavior. This complexity necessitates integrated, multidisciplinary, and comprehensive models of care. A new and interesting complementary approach is represented by “Dance Well”, i.e., an artistic, multisensory dance intervention based on art and music. This pilot study aims to evaluate the efficacy, feasibility, and safety of a 4-week Dance Well (DW) program in PD patients in early–medium disease stages. **Methods**: Twenty-four PD patients (H&Y ≤ 3; MoCA score ≥ 13.8) were enrolled and randomly allocated into two groups, both with a twice-per-week frequency and lasting 4 weeks: one group underwent the DW program, while the other underwent conventional physiotherapy (CPT). Demographic, biometric, and clinical data were collected. To study the treatment effect, motor (Unified PD Rating Scale-III, Timed Up and Go Test, Berg Balance Scale, 6-Minute Walk Test, and Falls Efficacy Scale), cognitive (Corsi Supra Span Test, Trail Making Test A and B-A), and emotional–motivational (Beck Depression Inventory, State-Trait Anxiety Inventory, Apathy Evaluation Scale) outcome measures were assessed, before and after the rehabilitation program. To study treatment compliance and safety, the number of dropouts and any adverse events (such as injuries and falls) were recorded. **Results**: All outcome measures improved in both groups. The percentage of improvement in outcome measures assessing attention and motivation was greater in the DW group. No dropouts, falls, or injuries occurred. **Conclusions**: In the early–medium stages of PD, DW could be considered a feasible and safe complementary treatment, useful in improving motor, cognitive, and emotional–motivational symptoms.

## 1. Introduction

In the novel *The Idiot*, Fyodor Dostoyevsky wrote the famous sentence, “beauty will save the world”. This sentence illustrates the power of art to influence the human brain. It was 1869, and nothing was known about neuroplasticity and the role of artistic experiences, such as dance, music, and visual arts in modulating this phenomenon. Nowadays, the knowledge of neuroplasticity, i.e., the power of the brain to adapt its networks and connections in response to ever-changing conditions, represents one of the columns of rehabilitation practice in neurology, including the case of neurodegenerative diseases, such as Parkinson’s disease (PD) [1,2].

PD is a progressive, neurodegenerative motor behavior disorder associated with poor mobility, gait difficulties, postural instability with falls, cognitive and motivational problems, and reduced quality of life. Evidence suggests that physical activity and motor–cognitive rehabilitation can improve mobility, balance, posture, cognition, mood, and quality of life in PD [3,4,5,6,7,8,9,10,11]. Despite the proven effectiveness, access to rehabilitative treatments and facilities may not always be guaranteed for both economic and organizational reasons. Further, adherence to exercise and physical activity programs can be challenging for some people with PD in the long term [12]. Such complementary, enjoyable interventions could address, at least in part, these relevant practical problems [13,14,15].

Among these, artistic experiences that combine physical activity with the principles of motor–cognitive rehabilitation—such as music, visual arts, and dance—could benefit PD patients by enhancing motor skills and cognitive functions, while also providing emotional benefits and opportunities for social interaction [13,16,17,18]. Dancing in various musical contexts (e.g., Irish set dancing, Argentine tango, ballet, ballroom dancing) has been reported to positively affect mobility, gait, balance, fall rates, daily living, and quality of life in PD [19,20,21,22].

Music and dance may promote the reorganization of brain networks at various motor, cognitive, and motivational levels by positively influencing entrainment, rhythm, motor coordination, motivation, and emotion in PD patients [23,24]. Further, art therapy has been shown to enhance overall visual–cognitive skills and visual exploration strategies, as well as general motor function, in patients with PD [25].

On this basis, in 2010, in an attempt to merge dance, music, and art, the “Dance Well” (DW) project (https://dancewell.eu/en/, accessed on 28 March 2025) was implemented in Bassano del Grappa (Italy). One of the cornerstones of DW relies on the prerequisite of dancing in the midst of artistic places blessed by beauty, such as museums, theatres, historical buildings, and galleries. The project was born as a multisensory intervention focused on improvisation and was conceived with the idea of immersing the patients into the beauty of music and visual arts for transforming their movements into dance. In DW classes, teachers refer to contemporary dance, and the practice is intended to guide the patient toward greater awareness of their motor control. Therefore, the DW cornerstone is to train movement through facilitation strategies favored by the musical–artistic context. This allows the patient to integrate their movements within an action context that, by promoting the translation of simple movements into “dancing” gestures, favors improvement in execution speed, amplitude, rhythm, and movement control. Incorporating the dancing protocol into an artistic context aims to maximize the visuospatial functions.

In this sense, DW has been specifically designed for people with early to moderately severe PD: specifically, it could be considered a rehabilitative strategy for guiding the re-learning of adequate movements by incorporating aerobic exercise and multiple cognitive stimuli (including rhythmic auditory and visual cues) to promote and reinforce the motor–cognitive integration [1,9,26]. The components of DW are relatively simple and easy to learn and perform for people with compromised mobility. The intervention is also enjoyable and engaging, as reported for other genres of dance [19,20,21,22].

Despite the growing enthusiasm for this intervention, unsolved questions remain: (a) Is DW safe and feasible for PD patients? (b) Is DW effective in ameliorating motor, cognitive, and emotional–motivational symptoms in PD patients, at least as much as CPT?

Therefore, as a prelude to a large-scale multicenter randomized controlled trial (RCT), we conducted a pilot study to explore the effectiveness, feasibility, and safety of the DW intervention for people with PD.

## 2. Materials and Methods

### 2.1. Design

From January to May 2022, we conducted a single-blind, parallel-group randomized controlled trial (RCT) to address the DW efficacy (as primary outcome), feasibility, and safety (secondary outcomes). The study was approved by the local ethics committee (EC Ausl Berica n.002-04/11/2021) and was in accordance with the Code of Ethics of the World Medical Association (Declaration of Helsinki, 1967). All participants signed an informed written consent form for the use of their clinical data for scientific purposes. This trial was registered on ClinicalTrials.gov website (NCT06896123).

### 2.2. Participants

Twenty-four patients were consecutively enrolled at the Villa Margherita Fresco Parkinson Center (Arcugnano, Italy) by neurologists with experience in movement disorders. Inclusion criteria were as follows: (a) diagnosis of PD according to the UK Parkinson’s Disease Society Brain Bank clinical diagnostic criteria [27]; (b) rate score 0–3 on the modified Hoehn and Yahr (H&Y) scale [28]. Exclusion criteria were as follows: (a) other neurological or psychiatric diseases other than PD; (b) serious visual and/or auditory deficits; (c) orthopedic–muscular and or co-morbid medical conditions precluding participation in dance; (d) cognitive decline (MoCA score < 13.8) [29].

Patients had not been exposed to any dance or physical interventions in the 12 months prior to the enrollment.

The following demographic, biometric, and clinical data were collected for each participant: age, gender, body mass index (BMI), weight and height, Hoehn and Yahr (H&Y) stage, disease duration, and PD medications (expressed as levodopa equivalent daily dose (LEDD)) [30].

### 2.3. Randomization

Participants were randomized to undergo a program of DW (DW group n = 12) or CPT (CPT group n = 12). We first stratified participants based on their H&Y stage. Within each H&Y group, we used blocks to randomize participants, assigning them to treatment groups in blocks of 2 to balance the number of participants in each treatment group within each stratum. We used computer-generated number sequences to randomly assign participants within each block to the treatment groups, ensuring that the allocation is unbiased and reproducible. By combining stratification and blocking, we aimed to achieve a balanced allocation of participants across treatment groups, considering the variability in disease severity as measured by the H&Y stage.

### 2.4. Intervention

Participants in the DW group received dance classes twice a week for four weeks. The dance studio was located in an artistic, historical building at Villa Margherita (Arcugnagno, Italy). The classes were held by two dancing teachers from the Dance Well Team in Bassano, Italy. A DW teacher was trained at the Fresco Parkinson Institute personnel regarding symptoms, therapies, and rehabilitation strategies in PD. Each DW dancing session was held in groups, lasted 1 h, and included a preliminary “warm-up phase” (10 min), a 40-min “dancing” phase, and a “cool-down” phase (10 min). All the phases were accompanied by music. The warm-up phase consisted of stretching and breathing exercises. During the next 40 min phase, patients were asked to put their attention on the surrounding acoustic, musical, and visual environment and encouraged to imagine, improvise, and emulate rhythmic and ample movements. Specifically, the DW teacher proposes the following: (i) proprioceptive exercises to enhance body awareness; (ii) exercises to be focused on step rhythmicity; (iii) exercises for training coordination, turning, balance, and postural control. The final, cool-down phase included relaxation exercises. Participants in the CPT group received 60 min conventional physiotherapy twice a week for four weeks. CPT was carried out by specialized physiotherapists. The protocol was designed in accordance with the KNGF guidelines for physical therapy in PD [31]. Each PT session was held individually, lasted 1 h, and included a preliminary “warm-up” phase (10 min), a central 40-min phase, and a “cool-down” phase (10 min). The warm-up phase consisted of stretching, and breathing exercises. The central phase was based on muscle strengthening, balance and postural control exercises, and gait training. The final, cool-down phase included relaxation exercises.

### 2.5. Outcome Measures Assessment

The outcome assessment included scales and tests to evaluate various clinical, motor, cognitive, and affective aspects. All outcome measure evaluations were performed by trained assessors blinded to group allocation at two time points: at T0 (enrollment) and T1 (end of the treatment—four weeks after enrollment). The clinical evaluation was conducted by a trained neurologist. The motor assessment was conducted by a physical therapist with experience in movement disorders, while the cognitive and affective assessments were carried out by an expert neuropsychologist.

The primary clinical outcome was Part III of the Unified PD Rating Scale (UPDRS), which aims to quantify motor impairment. It consists of 18 items that assess various aspects of motor function in individuals with PD, specifically speech, facial expression, tremor at rest, action or postural tremor, rigidity, finger taps, hand movements, rapid alternating movements of hands, leg agility, arising from a chair, posture, gait, postural stability, and body bradykinesia and hypokinesia. As secondary outcome measures, we used the following motor, cognitive, and emotional–motivational scores: (1) the Berg Balance Scale (BBS), the 6-Minute Walk Test (6MWT), the Timed Up and Go test (TUG), the Falls Efficacy Scale (FES); (2) the Corsi Supra Span Test (CSS), the Trail Making Test (TMT) A and B; (3) the Beck Depression Inventory (BDI), the State-Trait Anxiety Inventory (STAI-I), and the Apathy Evaluation Scale (AES).

BBS is a widely used tool designed to assess static balance and fall risk in adults. It consists of 14 tasks, each scored on a 5-point scale (0–4), with 0 indicating the lowest level of function and 4 the highest. Tasks include activities like sitting to standing, standing unsupported, reaching forward, and turning 360 degrees. It typically takes 15–20 min to administer.

6MWT is used to assess aerobic capacity and endurance by measuring the distance a person covers in six minutes while walking as far as possible on a flat, straight surface. Participants are encouraged to walk back and forth along a marked walkway, with encouragement provided at specific intervals.

TUG is used to evaluate mobility, balance, walking ability, and fall risk. Participants are asked to sit in a chair, stand up upon command, walk 3 m (10 feet), turn around, walk back to the chair, and sit down. The time taken to complete the task is recorded in seconds. A longer time indicates a greater risk of falling.

FES is used to assess the fear of falling. The scale consists of 16 items, each rated on a 4-point Likert scale (1–4), with 1 indicating no concern and 4 indicating severe concern. Questions regarding fear while cleaning the house, getting dressed, preparing meals, taking a bath, going to the shop, and walking on uneven surfaces are included. The total score ranges from 16 to 64, with higher scores indicating greater fear of falling.

CSS evaluates visuospatial working memory and learning processes for various types of information, surpassing the short-term memory span. Participants are positioned in front of a board with nine identical blocks arranged on it. They are required to replicate a sequence of nine blocks tapped by the examiner. Through repeated trials, up to a maximum of 18 repetitions, participants can learn and recall the sequences. The overall score is the sum of the scores obtained for each correctly reproduced sequence.

TMT is a paper-and-pencil test designed to assess visual attention, divided attention, and task switching. In TMT A, participants are asked to connect 25 numbered circles in ascending order, as quickly as possible. In TMT-B, subjects should connect 25 circles, alternating between numbers and letters (e.g., 1-A; 2-B), as quickly as possible. The time taken to complete the tasks is recorded in seconds. The B–A score represents the subtraction among TMT B and TMT A scores.

Beck Depression Inventory (BDI) is a self-administered questionnaire designed to measure depressive symptoms. It consists of 21 items, each of them item corresponds to a symptom of depression. Each item is rated on a 4-point scale (0–3). The total score ranges from 0 to 63, with higher scores indicating more severe depression.

State-Trait Anxiety Inventory Part I (STAI-I) is a self-administered questionnaire designed to measure state anxiety. It consists of 20 items assessing how one feels at the evaluation moment. Each item is rated on a 4-point scale, with higher scores indicating greater anxiety.

Apathy Evaluation Scale (AES), is a self-administered questionnaire, aimed to measure the level of apathy in individuals. It consists of 18 items (each of them rated on a 4-point scale), assessing behavioral, cognitive, and emotional aspects of goal-directed behavior. The total score ranges from 18 to 72, with higher scores indicating greater apathy.

### 2.6. Feasibility and Safety Evaluation

Any adverse events (such as injuries and falls) and the dropout number were recorded to evaluate the feasibility and safety of the treatments.

### 2.7. Statistical Analysis

Statistical analysis was performed using IBM SPSS Statistics for Windows (Version 25.0. IBM Corp, Armonk, NY, USA). Numerical data were expressed as means with standard deviations (mean ± SD) for all parameters. For all outcome measures, the data distribution was evaluated, applying the Kolmogorov–Smirnov and Shapiro–Wilk normality tests, together with the Levene test. The independent samples *t*-test was used to assess differences between groups on demographic, biometric, and clinical data as well as effectiveness data, when appropriate. For outcome measures with a normal distribution, we applied repeated measures ANOVA (RM-ANOVA) and post hoc analysis, when appropriate.

For outcome measures with a non-normal distribution, we applied the Kruskal–Wallis test for between-group comparisons and the Wilcoxon test for within-group comparisons. In order to deeply study the effectiveness, we calculated the percentage of improvement, using the following formula:

*Percentage of improvement* (*PoI*): [(*scoreT1* − *scoreT0*)/*score T0*] × 100

Regardless of the improvement direction of each outcome measure, all PoIs were reported as absolute values. For each outcome measure PoI, a one-way ANOVA was conducted to analyze the differences between groups. The PoI values for motor, cognitive, and emotional–motivational groups of outcome measures were obtained by averaging the PoI of each subgroup of outcome measures.

To control for the risk of Type I errors due to multiple comparisons, we applied the Bonferroni correction, setting a *p*-value of ≤0.0045 as significant.

## 3. Results

### 3.1. Participants

Table 1 shows the baseline features for both groups: they were comparable for all demographic, clinical, and biometric data. No between-group differences were found for key features such as height, weight, PD duration, H&Y stage, and MoCA scores.

The Wilcoxon test for within-subject was applied for analyzing UPDRS-III scores, showing improvement in both groups from T0 to T1 (DW group: Z = −3.062, *p* = 0.002; CPT group: Z = −3.036, *p* = 0.002). The Kruskal–Wallis test was applied for between-subject analysis: no differences were observed between groups, indicating the same trend from T0 to T1 (H_pre_ = 0.406; H_post_ = 0.13; *p* = 0.908).

We conducted a non-parametric analysis for TUG as well, obtaining similar results within subjects (both *p* = 0.002) and no significant differences between subjects (H_pre_: 0.608; H_post_ = 4.452; *p* = 0.035).

A repeated measures ANOVA was conducted to examine the effect of time and the interaction between time and group on 6MWT, BBS, and FES scores, which were the motor outcomes.

For 6MWT scores, there was a significant effect of time, F(1, 22) = 58.721, *p* < 0.001, η^2^ = 0.727. The interaction between time and groups was not significant, F(1, 22) = 0.015, *p* = 0.903, η^2^ = 0.001. There was no significant effect of groups, F(1, 22) = 1.296, *p* = 0.267.

For BBS scores, there was a significant effect of time, F(1, 22) = 75.133, *p* < 0.001, η^2^ = 0.774. The interaction between time and groups was not significant, F(1, 22) = 0.271, *p* = 0.608, η^2^ = 0.012. There was no significant effect of groups, F(1, 22) = 1.092, *p* = 0.307.

For FES scores, there was a significant effect of time, F(1, 22) = 70.912, *p* < 0.001, η^2^ = 0.763. The interaction between time and groups was not significant, F(1, 22) = 0.646, *p* = 0.430, η^2^ = 0.029. There was no significant effect of groups, F(1, 22) = 0.028, *p* = 0.868.

A repeated measures ANOVA was also conducted to examine the effect of time and the interaction between time and groups on CSST, TMT A, TMT B–A, which were the cognitive outcomes, and BDI, STAI-I, and AES, which were affective outcomes.

For CSST scores, there was a significant effect of time, F(1, 22) = 55.029, *p* < 0.001, η^2^ = 0.714. The interaction between time and groups was significant, F(1, 22) = 46.205, *p* < 0.001, η^2^ = 0.677. There was no significant effect of groups, F(1, 22) = 1.150, *p* = 0.295.

For TMT A scores, there was a significant effect of time, F(1, 22) = 39.725, *p* < 0.001, η^2^ = 0.644. The interaction between time and groups was significant, F(1, 22) = 22.968, *p* < 0.001, η^2^ = 0.511. There was no significant effect of groups, F(1, 22) = 5.424, *p* = 0.029.

For TMT B–A scores, there was a significant effect of time, F(1, 22) = 28.396, *p* < 0.001, η^2^ = 0.563. The interaction between time and groups was significant, F(1, 22) = 21.045, *p* < 0.001, η^2^ = 0.489. There was no significant effect of groups, F(1, 22) = 2.845, *p* = 0.106.

For BDI scores, there was a significant effect of time, F(1, 23) = 46.991, *p* < 0.001, η^2^ = 0.671. The interaction between time and groups was significant, F(1, 22) = 13.530, *p* = 0.001, η^2^ = 0.381. There was no significant effect of groups, F(1, 22) = 0.551, *p* = 0.466.

For STAI I scores, there was a significant effect of time, F(1, 22) = 77.807, *p* < 0.001, η^2^ = 0.780. The interaction between time and groups was significant, F(1, 22) = 40.366, *p* < 0.001, η^2^ = 0.647. There was no significant effect of groups, F(1, 22) = 6.136, *p* = 0.021.

For AES scores, there was a significant effect of time, F(1, 22) = 58.495, *p* < 0.001, η^2^ = 0.727. The interaction between time and groups was significant, F(1, 22) = 42.137, *p* < 0.001, η^2^ = 0.657. There was a significant effect of groups, F(1, 22) = 11.722, *p* = 0.002.

**Figure 1:**
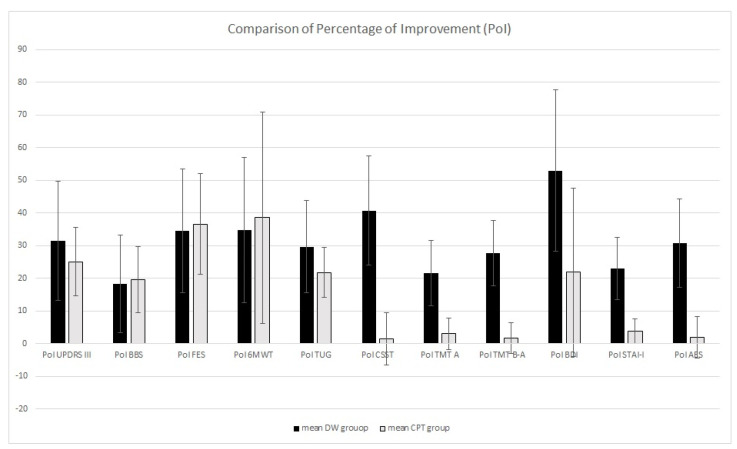
Comparison of percentage of improvement between the DW group and the CPT group.

After the PoI calculation, the one-way ANOVA showed no differences in PoI for UPDRS-III, BBS, 6MWT, and TUG. Otherwise, PoI in CSS, TMT A and B–A, BDI, STAI-I, and AES differed between groups: the DW group showed higher PoI (see Table 2 and Figure 1).

### 3.2. Feasibility and Safety

All intervention sessions were fully administered in both the DW and CPT groups. There was no significant difference between groups in the time spent in therapy: a mean of 480 min for both groups. No dropout occurred: all 24 patients concluded the rehabilitation treatment. Over the 4-week treatment period, one participant in the CPT group experienced an incidental, non-injurious fall. No other adverse events were recorded in the CPT group. No adverse events were recorded in the DW group.

## 4. Discussion

This is the first pilot study aimed at analyzing the effectiveness, feasibility, and safety of DW as a complementary rehabilitation treatment for PD.

DW represents an intriguing artistic, multisensory approach, as it integrates visual art, music, and dance. DW encourages patients to improvise movements and engage with the rhythm of music while socializing and immersing themselves in artistic environments, such as museums, galleries, theatres, and historic buildings.

This pilot study indicates that a 4-week DW program yields motor benefits comparable to those obtained with a 4-week CPT. This is likely due to the use of the musical rhythm that could influence the motor system in PD patients [32,33]. It is well known that the gait problems in PD, such as asymmetrical steps, short stride length, high cadence, and difficulty in gait initiation, turning, and stopping [34], mainly depend on the dysfunction of putamen in controlling the gait pacing [35]. One potential method to overcome putamen dysfunction could be represented by rhythmic cues that may be used as an ‘internal clock’ to facilitate synchrony of movements. Indeed, while self-initiated or self-paced movements are impaired in PD patients [36], externally paced movements (in response to either a tone or a visual cue) do not show such severe impairments [37]. For these reasons, extrinsic cues could facilitate movement [38] and may provide input for sequential movements by reducing the reliance on deficient automatized processes [39]. This is due to the hyperactivity of the cerebellum following the dysfunction of the basal ganglia. In PD, increased activity in the cerebellum compensates for the damaged basal ganglia–cortical pathways [40]. The cerebellum controls both entrainment (i.e., the capability to align movements with periodic external stimuli) and auditory–motor synchronization by monitoring rhythmic patterns and adjusting behavior to changing *tempos* [33,41]. This sensory–motor coupling, in which auditory information drives motor action, is not affected by the disease process at least in the early–mild disease stages. Therefore, rhythmic music may drive improvements in parameters such as speed, cadence, and stride length, either by bypassing or facilitating the impaired basal ganglia–cortical activity via the cerebellum [9,40].

While dancing, a rhythmically structured sound pattern generates an anticipatory template of a time sequence that may facilitate movement by enabling the timing of muscle activation. This happens because sounds can exert an influence on the motor pathway via reticulospinal connections, which prime and alter the timing of spinal motor neuron activity [40,42]. These latter considerations could help explain the improvement we found in motricity, balance, and coordination following DW.

Notably, in comparison with CPT, we found greater improvements in visual attention (studied with the TMT A) in the DW group. More generally, the PoI in visual functions is greater in the DW group than in the CPT group, exceeding 20% compared to the baseline values. Difficulties in visuospatial functions are detectable even in early PD [43,44]. Referring to normative data for the Italian population, our patients showed average scores falling in the borderline range for both the CSST and TMT A. These difficulties could affect visuospatial exploration and visuomotor integration, thereby exacerbating gait alterations and increasing the risk of falls, which negatively impacts numerous daily living activities. Previous studies have highlighted how specific motor–cognitive rehabilitation treatments are able to improve cognitive functions [1,2,9,26,45,46,47]. Parallelly, Cucca et al. [25] observed that an art rehabilitation program (twenty sessions lasting approximately 90 min each, twice a week, for ten consecutive weeks) led to improvements in visual functions and increased functional connectivity in cortical areas V1 and V2 in PD patients. The greater improvements in visual functions obtained in the DW group suggest that the artistic environment in which the dance session takes place probably serves as a salient facilitator that stimulates visual engagement, favors the elaboration of visual stimuli, and, consequently, enhances visuomotor integration. Probably, dancing within such artistic contexts, by promoting visuomotor integrations, represents the main additional value of DW in comparison to other forms of dance used for PD.

Interestingly, all the scales evaluating the emotional–motivational aspects showed greater PoI in the DW group compared to the CPT group, increasing the initial scores by at least 20%. It is important to highlight that the average scores for the BDI and AES were lower than the cut-off scores for depression and apathy. Conversely, the average STAI-Y score was higher, indicating elevated anxiety levels in our population. Despite the limited data about the efficacy of dance therapy on emotional and motivational symptoms [48], it is arguable that the DW program, by stimulating creativity and socialization within artistic environments and in the presence of relaxing music, could be effective for many affective problems in PD.

This pilot study presents several limitations that should be considered when interpreting the results. Firstly, the small sample size hinders our ability to draw definitive conclusions about the efficacy of DW. Larger trials will be necessary to confirm these findings, ensuring greater statistical power and more reliable estimates and allowing us to avoid Type I errors, which can occur when multiple comparisons are made with a small sample size. Based on the discharge SD and the estimation of clinically significant minimal effect sizes, we calculated the sample sizes for a future larger trial. We aimed to detect an effect size of 0.8, accounting for a 20% dropout rate, assuming two groups of equal size and a two-tailed significance threshold (alpha = 0.05). This method estimates the minimal clinically important difference (MCID). Therefore, at least 63 participants per group are recommended for a future trial, allowing for small levels of non-compliance.

Further, we studied only the immediate post-treatment effect. Different complementary physical interventions (such as aquatic therapy, yoga, tango, tai chi, and Qigong) present notable effects that usually decay within a given period after the end of rehabilitation [49,50,51,52,53]; therefore, a longitudinal follow-up would be of interest to determine whether and for how long DW benefits persist over time, or if DW provides a distinctive long-lasting effect compared to CPT or other complementary approaches.

These results should also be interpreted considering the significant impact that music and engagement in artistic activities can have on patients beyond the practical treatment effect. It has been suggested that music listening, viewing artwork, and creative arts have specific efficacy in enhancing mood and reducing negative mood states, particularly anxiety [54,55,56]. In this regard, it cannot be excluded that art-related elements, such as music and art, may have influenced the results through a placebo effect [57]. The placebo effect embodies complex and distinct psychoneurobiological phenomena where behavioral, neurophysiological, perceptive, and cognitive changes occur during therapy [58]. Given that music and art can play a significant role in creating a positive and engaging environment, their presence in rehabilitative settings may maximize placebo effects by leveraging the psychological and emotional benefits associated with artistic engagement and enhancing therapeutical efficacy [59]. In PD, when patients experience placebo-induced improvements, a large amount of dopamine is released in the dorsal motor striatum, suggesting a relationship between the amount of dorsal striatal dopamine release and clinical benefit [60]. Despite these suggestions, this study cannot provide indications about whether and to what extent the placebo effect works in DW.

Finally, we did not compare the treatments in terms of intensity, metabolic consumption, or amount of aerobic activity. Future studies are needed to clarify the rehabilitation-related dose effect within the same treatments and among conventional and complementary approaches.

## 5. Conclusions

DW is an artistic, multisensory dance intervention performed in artistic settings. Based on the integration of multiple stimuli, the DW components are relatively simple and easy to learn and perform for people with compromised mobility. In this pilot study, we found that DW is feasible and safe for PD patients in early–medium disease stages. Although the short duration and small sample size of this pilot study do not allow us to draw definitive conclusions, DW appears to be a helpful complementary treatment for improving motor, cognitive, and emotional–motivational symptoms in the early–medium stages of PD.

## Figures and Tables

**Table 1:** Demographic, clinical, and biometric data.

	DW Group	CPT Group	
Number	12	12	
Male (%)	50	50	
	Mean ± SD	Mean ± SD	*p*-Value
Education (years)	9.25 ± 4.789	8.42 ± 3.260	0.623
Disease Duration (years)	8.08 ± 4.699	8.92 ± 4.582	0.664
Age at PD Onset	62.50 ± 4.543	62.08 ± 2.193	0.777
LEDD (mg equivalents)	683.00 ± 547.715	792.17 ± 463.076	0.603
Weight (kg)	72.50 ± 13.548	73.50 ± 9.849	0.838
Height (cm)	171.17 ± 8.851	172.00 ± 10.189	0.833

LEDD: levodopa equivalent daily dose; DW: Dance Well; CPT: conventional physiotherapy.

**Table 2:** Outcome measures percentage of improvements (PoI).

	Mean SD	Mean SD	F	*p*-Value
	DW Group	CPT Group		
% of Motor Improvements				
PoI UPDRS III	31.44 ± 18.21	25.08 ± 10.47	1.102	0.305
PoI BBS	18.27 ± 14.98	19.57 ± 10.11	0.062	0.806
PoI FES	34.56 ± 18.93	36.64 ± 15.44	0.087	0.771
PoI 6MWT	34.85 ± 22.26	38.58 ± 32.45	0.108	0.746
PoI TUG	29.67 ± 14.06	21.80 ± 7.72	2.888	0.103
% of Cognitive Improvements				
PoI CSST	40.73 ± 16.64	1.44 ± 8.09	54.104	0.000
PoI TMTA	21.63 ± 9.97	2.97 ± 4.77	34.227	0.000
PoI TMT B-A	27.70 ± 11.29	1.70 ± 5.42	51.733	0.000
% of Emotional–Motivational Improvements				
PoI BDI	52.96 ± 24.78	21.81 ± 25.84	9.088	0.006
PoI STAI-1	22.96 ± 9.55	3.69 ± 3.79	42.265	0.000
PoI AES	30.75 ± 13.53	1.98 ± 6.36	44.479	0.000

PoI: percentage of improvement; UPDRS III: Unified PD Rating Scale Part III; BBS: Berg Balance Scale; 6MWT: 6-Minute Walk Test; TUG: Timed Up and Go Test; CSST: Corsi Supra Span Test; TMT: Trail Making Test; BDI: Beck Depression Inventory; STAI-I: State-Trait Anxiety Inventory Part I; AES: Apathy Evaluation Scale.

## Data Availability

Data supporting the reported results can be provided upon request to the corresponding author due to the procedural policies of the neurorehabilitation ward where the study was conducted.

## Abbreviations

The following abbreviations are used in this manuscript:

PD	Parkinson’s disease
DW	Dance Well
H&Y	Hoehn and Yahr
CPT	conventional physiotherapy
UPDRS	Unified Parkinson’s Disease Rating Scale
TUG	Timed Up and Go Test
BBS	Berg Balance Scale
6MWT	6-Minute Walk Test
FES	Falls Efficacy Scale
CSS	Corsi Supra Span Test
TMT	Trail Making Test
BDI	Beck Depression Inventory
AES	Apathy Evaluation Scale
MoCA	Montreal Cognitive Assessment
PoI	percentage of improvement
MCDI	minimal clinically important difference
SD	standard deviation
STAI	State-Trait Anxiety Inventory
EC	ethics committee
